# Collaborative stepped care for anxiety disorders in primary care: aims and design of a randomized controlled trial

**DOI:** 10.1186/1472-6963-9-159

**Published:** 2009-09-08

**Authors:** Anna DT Muntingh, Christina M van der Feltz-Cornelis, Harm WJ van Marwijk, Philip Spinhoven, Willem JJ Assendelft, Margot WM de Waal, Leona Hakkaart-van Roijen, Herman J Adèr, Anton JLM van Balkom

**Affiliations:** 1 Netherlands Institute of Mental Health and Addiction (Trimbos-institute), Utrecht, the Netherlands; 2 The EMGO Institute for health and care research (EMGO+), Amsterdam, the Netherlands; 3 Department of General Practice, VU University Medical Centre, Amsterdam, the Netherlands; 4 Department of Psychiatry, VU University Medical Centre, Amsterdam, the Netherlands; 5 Department of Psychology, Leiden University, Leiden, the Netherlands; 6 Department of Public Health and Primary Care of the Leiden University Medical Centre, Leiden, the Netherlands; 7 Institute for Medical Technology Assessment, Erasmus University, Rotterdam, the Netherlands; 8 Johannes van Kessel Advising, Huizen, the Netherlands

## Abstract

**Background:**

Panic disorder (PD) and generalized anxiety disorder (GAD) are two of the most disabling and costly anxiety disorders seen in primary care. However, treatment quality of these disorders in primary care generally falls beneath the standard of international guidelines. Collaborative stepped care is recommended for improving treatment of anxiety disorders, but cost-effectiveness of such an intervention has not yet been assessed in primary care. This article describes the aims and design of a study that is currently underway. The aim of this study is to evaluate effects and costs of a collaborative stepped care approach in the primary care setting for patients with PD and GAD compared with care as usual.

**Methods/design:**

The study is a two armed, cluster randomized controlled trial. Care managers and their primary care practices will be randomized to deliver either collaborative stepped care (CSC) or care as usual (CAU). In the CSC group a general practitioner, care manager and psychiatrist work together in a collaborative care framework. Stepped care is provided in three steps: 1) guided self-help, 2) cognitive behavioral therapy and 3) antidepressant medication. Primary care patients with a DSM-IV diagnosis of PD and/or GAD will be included. 134 completers are needed to attain sufficient power to show a clinically significant effect of 1/2 SD on the primary outcome measure, the Beck Anxiety Inventory (BAI). Data on anxiety symptoms, mental and physical health, quality of life, health resource use and productivity will be collected at baseline and after three, six, nine and twelve months.

**Discussion:**

It is hypothesized that the collaborative stepped care intervention will be more cost-effective than care as usual. The pragmatic design of this study will enable the researchers to evaluate what is possible in real clinical practice, rather than under ideal circumstances. Many requirements for a high quality trial are being met. Results of this study will contribute to treatment options for GAD and PD in the primary care setting. Results will become available in 2011.

**Trial registration:**

NTR1071

## Background

Anxiety disorders are a great burden for patients, the general health system and society as a whole. Patients having an anxiety disorder suffer from considerable disability and reduced quality of life [1]. In addition, anxiety disorders are associated with significant costs due to the use of health services and reduced productivity [2].

Of the anxiety disorders, panic disorder (PD) and generalized anxiety disorder (GAD) are the most disabling [3] and costly [4-6] anxiety disorders that are frequently seen in primary care. Research has indicated that four to seven percent of primary care attendees suffer from one or both of these anxiety disorders [7-10].

As the majority of these patients is only seen in primary care [6,11], this may be a convenient setting to treat these disorders. Treatment for PD and GAD can be highly effective [12,13]. In recent decades the evidence for the effectiveness of treatments for anxiety disorders has been reviewed and described in clinical guidelines for treatment, where cognitive behavioral therapy as well as prescription of antidepressants are considered as first choice of treatment for PD and GAD [14-16]. However, these guidelines are rarely adhered to in primary care. About one third of patients with an anxiety disorder treated in primary care receive appropriate treatment as defined by a minimal accordance with existing guidelines [6,17,18].

One of the reasons for the low quality of treatment is poor recognition of anxiety disorders. Even when compared to depression, the recognition rate of anxiety disorders is low, with about one third of anxiety disorder patients labeled as such by their general practitioner (GP) [19-21]. Several factors are involved in this low recognition rate, such as patients unwillingness or inability to discuss their anxiety problems with their GP [11,22,23] and limited knowledge of GPs about psychiatric disorders. Moreover, GPs frequently work under time pressure and perceive they have not enough time to enquire about emotional problems. In conclusion, competing demands of the patient, the GP and the primary care structure of acute episodic care make diagnosing mental health problems difficult [24].

Although ameliorating recognition of anxiety disorders is necessary [25], it is not sufficient for improving primary health care for these patients [26-28]. GPs often feel they do not have the necessary capabilities to treat these problems [23,29]. Moreover, the primary care system does not seem to be well organized for care for anxiety disorders [25]. As anxiety disorders often have a chronic nature [30], they make a poor fit with the acute disease model of primary care [10]. Therefore, several researchers have proposed to use a chronic care model to implement evidence based care into practice. The most promising of these strategies are based on Wagner's model of care for chronic diseases [31]. This model was originally developed to improve treatment for chronic diseases like diabetes. The strategies following Wagner's model involve collaborative disease management with a pivotal role for a "care manager", who coordinates care, works according to an evidence-based treatment protocol, monitors treatment response and actively follows the patient. This care manager usually is a non-physician professional, who works in close collaboration with the GP. Care manager and GP are further assisted by a specialist from secondary care. This model was adopted for use with mental disorders, with a nurse practitioner or a psychologist as care manager and a psychiatrist functioning as consultant specialist [32,33].

This collaborative care model has been tested extensively in the treatment of depression, showing robust positive results [34,35]. A few studies in the United States have investigated the effectiveness of collaborative care for anxiety disorders, especially PD [36-38] and GAD [37]. When compared to other strategies for improving care for anxiety disorders in ambulatory care, collaborative care seems to be the most effective [39]. In two of the studies described above a cost-effectiveness analysis was performed. In both studies, collaborative care was more effective than care as usual. Results regarding cost effectiveness were inconclusive, with collaborative care being either more or less costly than care as usual [40,41]. Researchers of these collaborative care trials [36] and international guidelines [15] recommend a stepped care approach for mental health care in primary care, with least invasive and costly interventions preceding more invasive and expensive forms of care. Such an approach may make collaborative care interventions more cost-effective.

This article describes the aims and methods of a randomized controlled trial to test the effectiveness of a collaborative stepped care intervention for PD and GAD in primary care in the Netherlands. Such a study is warranted for two reasons. First, there has been no study on the cost-effectiveness of a collaborative care intervention for anxiety disorders that includes a stepped care approach. Second, published studies about collaborative care for anxiety disorders all stem from the United States (US), where the collaborative care model was originally developed. As there are significant differences across health care systems in the US and in European countries [42], the results of the collaborative care studies might not be generalized to other countries without consideration. To fill this gap in research, we designed a collaborative stepped care intervention for GAD and PD in the primary care setting. The treatment algorithm is built up from three interventions that have separately been proven effective and feasible in the primary care setting [29,43]. The interventions consist of guided self help, cognitive behavioral therapy, and antidepressant medication [43]. Other elements of collaborative care include a trained care manager (a mental health practice nurse or psychologist) who coordinates care and provides psychological treatment, the availability of a consultant psychiatrist for advising GP and care manager, telephone follow-up by the care manager and monitoring of anxiety symptoms to evaluate treatment progress and outcome. Effects and costs of the interventions will be assessed and an economic evaluation will be performed to estimate cost-effectiveness and cost-utility of the intervention. All relevant costs to society associated with the burden of anxiety disorders will be taken into account. In accordance with the outcomes of similar previous studies, it is hypothesized that the collaborative stepped care intervention will be at least more effective and possibly less expensive than care as usual.

## Methods/Design

### Objectives

The primary aim of this randomized controlled trial (RCT) is to evaluate the effectiveness of collaborative stepped care (CSC) versus care as usual (CAU) in the treatment of panic disorder (PD) and generalized anxiety disorder (GAD) in primary care, with severity of anxiety symptoms as primary outcome measure. The secondary aim is to evaluate cost-effectiveness (costs of the intervention weighed against a reduction in anxiety symptoms) and to estimate cost-utility (costs of the intervention weighed against gained Quality Adjusted Life Years (QALY's)).

### Study design

The study design is a two-armed, cluster randomized, controlled trial.

### Time frame

This study was initiated in 2008 and will take three years. Results are expected in 2011.

### Recruitment of GPs, care managers and psychiatrists

The study is designed in cooperation with the Netherlands Institute of Mental Health and Addiction (Trimbos Institute), the Department of General Practice and Psychiatry of the VU University Medical Centre in Amsterdam and the Department of Public Health and Primary Care of the Leiden University Medical Centre. GPs in the Leiden region that are located in the region of a large mental health centre (*Rivierduinen*) will receive an invitation to participate in the study, after which a researcher (AM) will contact all practices by phone to recommend participation. Participating practices will be able to decide which professional (e.g. a psychologist, a mental health practice nurse or a social worker) will fulfill the role of care manager. If the practice does not have such a professional available, a mental health practice nurse working at the regional mental health centre will be available to work in the practice. Experienced psychiatrists working at the regional mental health centre will perform as consultant psychiatrists for the intervention practices.

### Randomization

**Figure 1:**
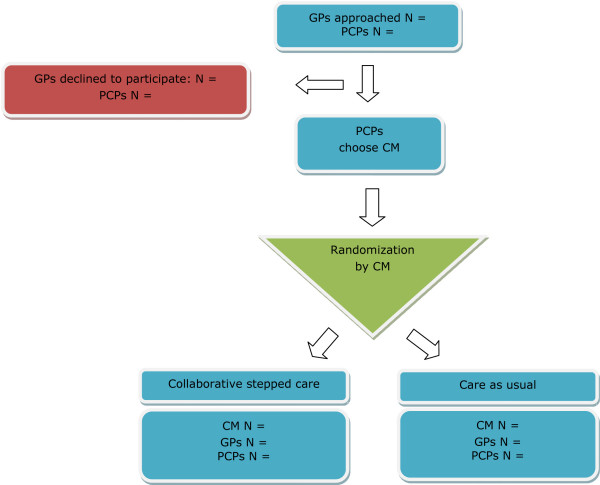
**Flowchart showing the recruitment and randomization of care managers and practices**. GP: general practitioner, PCP: primary care practice, CM: care manager.

Cluster randomization will be applied at the level of the care manager to minimize contamination of the effect [44]. Randomization will be performed using sequences obtained with an automated random sequence generation algorithm following a blocking scheme of variable length with allowance for restricted unbalance of at most three. Stratification will be on region, with six regions in total, which are based on working units of the regional mental health centre. The allocation sequences will be generated by an independent statistician (HA) in the manner described above. The care managers will be randomized and allocated to the intervention (CSC) or the control group (CAU). PCPs and GPs will be allocated to either CSC or CAU in accordance with the randomization status of their care manager. After randomization, neither care managers nor GPs will be blinded to group assignment. Figure 1 presents a flowchart of the recruitment and randomization procedure.

### Patient inclusion and exclusion criteria

Patients with a primary diagnosis of PD with or without agoraphobia and/or a primary diagnosis of GAD according to the criteria of the DSM IV [45] will be included in the study. Patients who are suicidal, suffer from dementia or other severe cognitive disorders, psychotic disorder, bipolar disorder, dependence on drugs or alcohol, or with an unstable severe medical condition as diagnosed by their GP or as assessed in a diagnostic interview will be excluded. Patients with insufficient knowledge of the Dutch language to fill out the questionnaires, patients who are already receiving intensive psychological treatment (>2 contacts per month with a psychologist or psychiatrist) and patients who are under 18 years of age will also be excluded from the study. For reasons of generalization, no other exclusion criteria are used. Having received treatment for anxiety problems in the past, using medication (e.g. antidepressants or benzodiazepines) or a diagnosis of co-morbid psychiatric and medical conditions (except for those described above) will not be reasons for exclusion.

### Recruitment of patients

Recruitment of patients will take place in two phases: a screening phase and a diagnostic phase. Patients will either be referred by their GP or will receive an invitation to participate based on a selection of the electronic medical record system of the GP. Patients are blinded for randomization status until they have returned the baseline questionnaire.

### Screening phase

GPs are able to refer a patient by handing a patient an information letter, an informed consent form and a short screening instrument: the Patient Health Questionnaire anxiety subscale (PHQ22) [46]. This measure has shown good psychometric properties for screening for anxiety disorders [46,47].

In cluster randomization, when dependent on referrals of GPs, a known problem is the inclusion of patients in the CAU group [48]. To diminish recruitment bias, referral by GPs is complemented with selection on basis of screening in this study. A number of patients will be selected from the electronic medical records of the GPs according to the following criteria: they are older than 18 years of age and had contact with their GP in the past four months for one of the following reasons: psychological or social problems, muscle or skeletal pain, fatigue, hyperventilation, fainting, stomach ache, complaints about functioning of the heart or head ache. These patients will receive an information letter, an informed consent form and the PHQ-22. Of patients who return the PHQ-22 and give informed consent, the score on the PHQ-22 will be calculated. Patients will be considered screen-positive if they answer affirmatively to the screening questions of the PHQ22 and list at least 4 symptoms for panic or at least 1 symptom for general anxiety [46]. For PD, threshold criteria will be used [49], where as for GAD the sub threshold criteria will be used to increase sensitivity [50]. Screen-positive patients will enter the diagnostic phase and will be contacted by telephone to perform a diagnostic interview.

### Diagnostic phase

**Figure 2:**
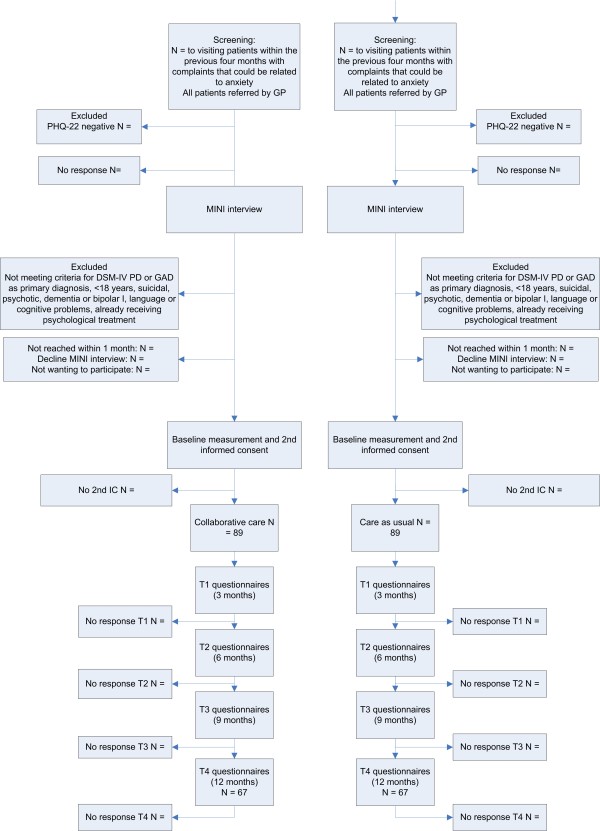
**Flowchart of participants**. GP: general practitioner, PCP: primary care practice, PHQ22: Patient Health Questionnaire anxiety sub-scale, MINI-interview: MINI-International Neuropsychiatric Interview.

Diagnostic interviews will be conducted by trained research assistants who will be blind to the randomization scheme. The MINI-PLUS International Neuropsychiatric Interview is a semi-structured interview that is often used for DSM-IV classification [51,52]. Telephone administered psychiatric interviews are found to have a high concordance with in-person interviews [53]. The interviewers will have the opportunity to consult a psychiatrist, who is also blind to randomization status, when they are uncertain of a diagnosis. Patients with a primary diagnosis of PD and/or GAD and who do not meet any of the exclusion criteria will receive a second information letter, baseline questionnaires and a second informed consent form. Patients will be offered the choice of a pen and paper version or an internet based version of the questionnaire. If the patient returns the baseline questionnaires and gives informed consent, the patient will be included in the study. Patients in the CSC group will be invited for a consultation with the care manager whereas patients in the CAU group will be advised to seek contact with their GP for treatment of their anxiety complaints. GPs in the control group will not be notified of the diagnosis of participating patients. Figure 2 shows a flowchart of participating patients.

### Sample size

The aim of the trial is to detect a clinically relevant difference of 0.5 SD (Cohen's effect size) of CSC versus CAU on the continuous measure of the Beck Anxiety Inventory (BAI) [54]. Sample size calculation is based on scores of 281 primary care patients in the multisite Netherlands Study of Depression and Anxiety (NESDA) [55] with a diagnosis of PD or GAD in the last six months. The mean BAI score in this sample was 16.94, with an SD of 10.49 (range 0-58). Hence, the expected difference between CSC and CAU is 6 points on the BAI. To demonstrate this difference with alpha = 0.05 and a power of 0.90, 64 cases per arm are needed ((1.96 + 1.28)^2 ^*10.49^2 ^*2)/6^2^). Since the average class size (n) is estimated to be 5 and the intraclass correlation coefficient (rho) is expected to be 0.01 [56,57], we apply an inflation factor of 1.04 (inflation factor = 1 + (n-1) × rho = 1 + 4 * 0.01 = 1.04) [58]. To be able to analyze 67 completers per arm and with an estimated 25% loss to follow-up, we aim to include 89 patients per arm.

### Intervention

#### Training

Care managers randomized to the intervention group will receive three days of training in the nature of anxiety disorders, collaborative care, the guided self-help intervention and cognitive behavioral therapy (CBT). These training sessions will be conducted by a psychologist (AM), the psychiatrist who developed the guided self help method [59] and two experienced cognitive behavioral therapists working at the regional mental health centre *Rivierduinen*.

GPs in the intervention group will receive three hours of training in the recognition of anxiety disorders, motivating patients for treatment, collaborative care and the medication algorithm. A psychiatrist (AvB), a GP (HvM) and a psychologist (AM) will provide this training.

Consultant psychiatrists will receive two hours of training in collaborative care, medication for PD and/or GAD and giving consultations in primary care. Two psychiatrists (AvB and CFC) and a psychologist (AM) will provide this training.

#### Treatment in the intervention group

##### 1. Collaborative stepped care

In accordance with the collaborative care model, care is provided by a team of the GP, the care manager, the patient and a consultant psychiatrist. The collaborative stepped care intervention is composed of four steps:

1) Guided self-help

2) Cognitive behavioral therapy (CBT)

3) Antidepressants according to a medication algorithm

4) Optimization of medication in primary care or referral to secondary care

After each step, progress is evaluated with the Beck Anxiety Inventory (BAI) [60]. The goal of the intervention is remission, according to the BAI score (See *7. Monitoring *for remission criteria). If a patient does not achieve criteria for remission after concluding a step, he or she proceeds with the next step. For example, when a patient does not achieve criteria for remission concluding the guided self-help program (step one), he or she is offered CBT treatment (step 2). In contrast, when a patient does achieve remission after the guided self-help program, he or she enters a program of relapse prevention.

**Figure 3:**
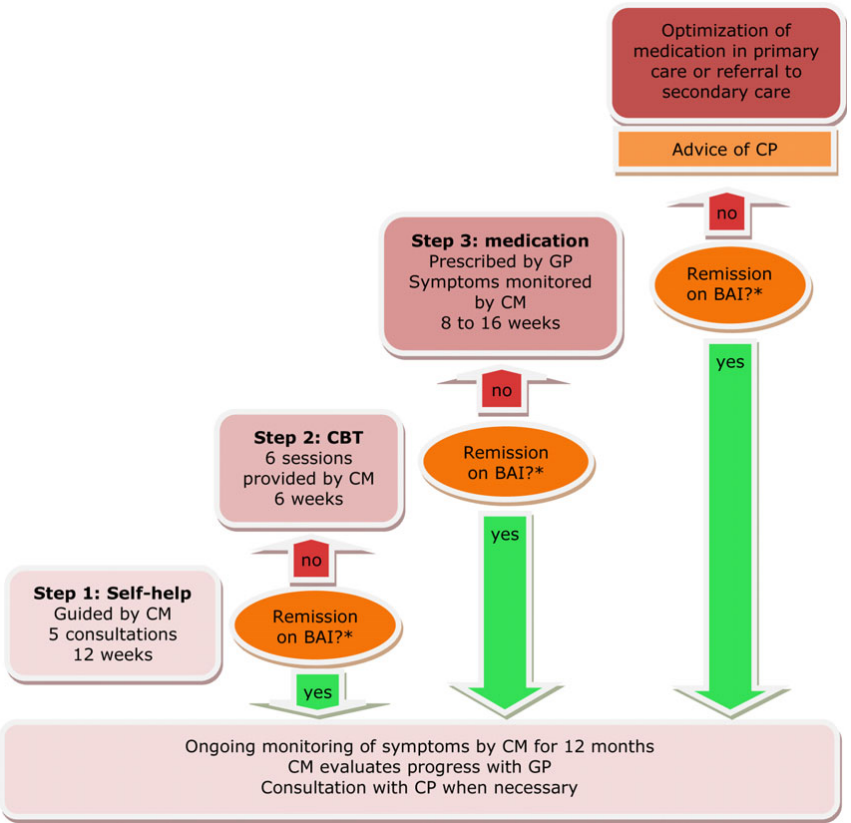
**Treatment algorithm**. *Remission is defined as a 50% reduction in score on the BAI plus a score of 11 or below. CM = care manager, GP = general practitioner, CP = consultant psychiatrist

The care manager coordinates care, delivers guided self-help and CBT and evaluates each step. The GP prescribes medication and evaluates progress with the care manager. The care manager as well as the GP can consult a consultant psychiatrist about treatment decisions. The active phase of the treatment lasts for at least 12 weeks and has a maximum of 34 weeks. Patients' adherence to the program is enhanced by contracting and active monitoring. Relapse prevention is provided by the care manager through monthly follow-up calls, until twelve months after the beginning of treatment. Figure 3 depicts the treatment algorithm.

##### 2. Contracting

When a patient is included in the study, he or she is invited for a first meeting with the care manager and GP. They shortly discuss the patients symptoms and explain the diagnosis. The patient is actively involved in the treatment plan by contracting. The patient receives a copy of the treatment plan.

##### 3. Improving adherence

Premature termination of treatment and diminished adherence to treatment are associated with poorer outcomes. Therefore, patient adherence is encouraged by psycho-education, goal setting and by frequent follow-up appointments in which both adherence and progress are evaluated. Provider adherence to the treatment protocol is encouraged by instructions from the researchers and newsletters, by frequent reporting about the care given, by recording of sessions and by regular supervision. Care managers attend a supervision group lead by a cognitive behavioral therapist every three weeks. They also have the opportunity to discuss problems and exchange experiences through an intranet forum.

##### 4. Guided self-help

Step one in the intervention is a guided self-help method based on cognitive behavioral principles [61]. This intervention was proven effective in a randomized controlled trial with patients with PD and/or GAD [29]. In twelve weeks, the patient works through a self-help manual with information about anxiety disorders, automatic thoughts, relaxation techniques and exposure in vivo [62]. Every chapter contains exercises for the patients to perform. In five short consultations spread over twelve weeks, the care manager informs the patient about the content of the manual, reinforces achievements and motivates the patient to continue. In addition, the patient is encouraged to find a "helper", a friend or a relative, who can help him or her perform exercises and support the patient to adhere to the program.

##### 5. Cognitive Behavioral Therapy (CBT)

CBT has been proven effective in numerous studies for both PD and GAD and is recommended as a first line treatment in international guidelines [63,64]. For this study, a short duration protocol was developed based on (inter)national guidelines and available manuals for the treatment of anxiety disorders with CBT [12,65,66].

A separate treatment protocol for PD and GAD is used, depending on the primary diagnosis of the patient. The CBT comprises a course of 6 sessions of 45 minutes provided by the care manager. All fundamental elements of CBT are represented in this protocol. For PD the main topics are psycho-education (e.g. the "cycle of panic"), registration of panic attacks, interoceptive exposure, recognition and modification of anxiety evoking automatic thoughts and behavioral experiments. The protocol for GAD focuses on psycho-education, recognition and modification of anxiety evoking automatic thoughts and meta-cognitions [67]. Both protocols employ homework assignments.

##### 6. Medication

GPs are encouraged to adhere to an antidepressant algorithm to optimize the prescription of antidepressants. The choice of recommended antidepressants was based on (inter)national guidelines. The algorithm includes time to titrate to daily dosages (2 weeks), time to respond (partially or remission, 4 weeks) and step-up criteria and methods (e.g. 'partial response: increase dose', 'no response: switch medication'). The care manager monitors adherence, adverse effects and response to treatment with the BAI. The GP and the care manager consult a psychiatrist when necessary. If the patient fulfils criteria for remission, medication treatment should be continued until twelve months after the initiation of medication treatment. If the patient does not respond or adhere to medication, the GP contacts the psychiatrist to discuss the options for further treatment. If further treatment in the primary care setting is not feasible, the patient may be referred to specialty mental health care.

##### 7. Monitoring

The care manager monitors anxiety symptoms with the BAI [60]. The goal of the intervention is remission, defined as a 50 percent reduction in score plus a score of 11 or below (see *Secondary outcome measures*). At the start of treatment the care manager administers the BAI and calculates a "target score" (remission) for each patient. The care manager then assesses the BAI at the end of step one and step two. During step three (medication), the care manager monitors symptoms more frequently: in week four and eight of medication use. If the patient switches medication this pattern is repeated.

##### 8. Relapse prevention

If a patient achieves remission after step one, two or three, relapse prevention is offered by the care manager. The care manager calls the patient every month, to assess anxiety symptoms with the BAI. If the BAI score of a remitted patient increases to a score of 12 or above on two consecutive measurements, the care manager consults the psychiatrist about the next step to be taken. The patient can "step up" to the next step (i.e. step 2: CBT or step 3: medication) or be referred to specialty mental health care. Relapse prevention lasts until one year after starting treatment.

##### 9. Referral to specialty mental health care

There can be several reasons for referring a patient to specialty mental health care: diagnostic uncertainty, complex psychosocial issues, poor response to treatment, patient preferences or emerging severe psychopathology. Referral to specialty mental health care is always discussed with the consultant psychiatrist.

#### Treatment in the control group

Half of the PCPs function as a control group. These GPs and care managers receive no training and they provide their usual care to their patients. There is a Dutch guideline available for all GPs about the treatment of anxiety disorders in primary care [68]. Care as usual comprises every form of care the GP is used to offer to his patient (e.g. watchful waiting, prescription of medication, referral to a mental health care professional or any other form of care the GP offers to his patient). The actual content of usual care will be assessed with the Scale for Medical Utilisation of Health Services [69].

### Data collection

Measurement will take place at baseline (T0), three (T1), six (T2), nine (T3) and twelve months (T4) after inclusion. The filled out pen and paper questionnaires will be processed anonymously by blinded research-assistants. The internet-questionnaires will be processed automatically.

### Outcome parameters

#### 1. Primary outcome measure

The severity of anxiety symptoms is measured with the Beck Anxiety Inventory (BAI) [60]. This measure lists 21 symptoms of anxiety like feeling hot, scared or nervous. Patients are instructed to rate how much each of these symptoms bothered them in the past week, including today. Each item can be rated on a 4 point Likert scale, ranging from 0 (Not at all) to 3 (Severely) yielding a maximum total score of 63 points. The instrument has good psychometric properties [70].

#### 2. Secondary outcome measure

##### Remission

As there is no standard "remission score" for the BAI, we calculated this score following the criteria of clinical significance of Jacobson & Truax [71]. These authors state that the best method to define recovery is to calculate the mean between the mean score of a population with the disorder and the mean score of a population without the disorder. We were able to derive these data of patients with or without PD or GAD from the NESDA study [55], resulting in a score of 11. Because the BAI score is not a part of the diagnostic procedure, it is expected that not every patient will score 11 or higher on the BAI. Therefore, we added the element of a 50 percent reduction in score to the definition of remission. In sum, remission is defined as a score of 11 or below, plus a 50% reduction in score.

#### 3. Additional outcome measures

##### Anxiety severity and impairment

Anxiety severity and impairment are measured by the Overall Anxiety Severity and Impairment Scale (OASIS) [72]. This scale was developed to measure severity of anxiety and impairment caused by anxiety across different anxiety disorders and showed good reliability and validity [73]. The scale consists of five questions about frequency of anxiety, severity of anxiety, avoidance, interference with tasks and interference of social relationships. The scale was translated in Dutch according to the forward-backward translation method.

##### Physical symptoms

Physical symptoms are measured by the Physical Symptoms Questionnaire (LKV: Lichamelijke Klachten Vragenlijst [74]), which assesses the number and intensity of functional somatic complaints a patient is experiencing. The Whitely Index (dimensional version [75,76]) measures attitudes about diseases (hypochondriasis). As these symptoms often co-occur with anxiety disorders, it is interesting to see whether these symptoms also decrease when treating the anxiety disorder.

##### Quality of life

Quality of life is a measure that allows comparison in different studies and (mental and physical) disorders. In this study, quality of life is assessed with the EuroQol (EQ-5D) [77] and the Short Form-36 (SF-36) [78], both validated instruments for measuring general health-related quality of life. The EQ-5D descriptive system consists of five dimensions (mobility, self-care, usual activities, pain/discomfort, and anxiety/depression), each with three levels (no problems, some problems, and extreme problems), thus defining 243 distinct health states. The SF-36 is an often used measure that assesses eight health concepts [78].

#### 4. Effect modifiers

##### Demographic variables

The following demographic variables are measured at baseline: age, gender, nationality and ethnicity, marital status, living conditions, education and work status.

##### Physical illness

Co-morbid physical illness is measured at baseline by means of a questionnaire developed by Statistics Netherlands (the CBS list), which lists 28 chronic conditions (e.g. diabetes type II and vascular disease). Chronic medical illness is found to be related to more severe symptoms at baseline, but not to a different treatment response in the treatment of anxiety disorders [79].

##### Depressive symptoms

Depressive symptoms are assessed with the depression-subscale of the Patient Health Questionnaire (PHQ9) [46,80], a brief and valid instrument which measures each of the DSM-IV criteria for major depressive disorder. The total score gives an indication about the severity of depressive symptoms. Depression is related to more severe symptoms at baseline and after treatment, but not to a different treatment response [81]. Depressive symptoms are assessed at all measurement points, to see whether depressive symptoms also reside when treating the anxiety disorder and to be able to detect a possible difference between PD and GAD.

##### Coping

The use of specific coping styles is measured by the Utrecht Coping List (UCL: Utrechtse Coping Lijst) [82]. This list assesses the frequency of using seven different coping styles: active coping, palliative reaction, avoidance, seeking social support, passive coping, expression of emotions and comforting thoughts. Coping styles are found to be related to outcome in mental disorders [83]. There is a debate whether coping styles are sensitive to change following treatment.

### Economic evaluation

The aim of this economic evaluation is to assess the cost effectiveness and to estimate cost utility of CSC compared to CAU. This will be done by relating the difference in direct medical costs per patient receiving CSC or CAU to the difference in terms of reduction in score on the BAI (cost-effectiveness) and *quality adjusted life years (QALY) *gained (cost-utility). This will yield a cost per unit of the BAI and per QALY estimate. QALY's will be estimated using the 'Dutch EQ-5D tariff', which is used to calculate utilities for EQ-5D health states for the cost utility analyses of Dutch health care programs and treatments [84]. The analyses will also be performed including productivity costs.

#### Medical costs

For calculating the total direct medical costs, the Trimbos/iMTA questionnaire for Costs associated with Psychiatric Illness (TiC-P) [85,86] is used. The Tic-P measures direct costs of medical treatment such as the number of contacts with the GP and multiple other care providers (e.g. medical specialists and paramedics) during the last three months. Medication use is measured during the last four weeks. The costs will be estimated in line with the Dutch guidelines for cost calculations in health care [87].

Reference unit prices of the corresponding health care services will be applied, and adjusted to the year of the study according to the consumer price index. Since the collaborative stepped care model is a new kind of intervention, a unit price per session is currently not available. To determine a reference price for this intervention a micro-costing study will be performed in at least three PCPs delivering the collaborative care intervention. Time for face-to-face contacts with the patient as well as indirect time per contact (e.g. mutual consultation contacts between GP and the care manager or the consultant psychiatrist) will be measured. For reasons of comparison the costs for a GP contact in the CAU study-arm will be measured applying a similar micro-costing methodology.

#### Productivity costs

For collecting data on productivity losses a short form of the Health and Labour questionnaire (SF-HQL) [88] is used. The SF-HLQ consists of three modules that measure productivity losses: absence from work, reduced efficiency at work and difficulties with job performance [89].

Productivity losses as measured by the SF-HQL are valued according to the average value added per worker by age and gender per day and per hour. If respondents indicate that they have been absent for the entire recall period, data will be collected from the time when the period of long-term absence started. This additional information will be used to value the production losses according to the "friction cost method" [90]. This method takes into account the economic circumstances that limit the losses of productivity to society, which are related to the fact that a formerly unemployed person may replace a person who becomes disabled.

### Statistical analyses

Intention-to-treat analysis will be performed by multilevel analysis with time as the first hierarchical level, patients as the second hierarchical level and care managers with their PCPs in the third level [91]. Possible confounding characteristics (e.g. age, gender or level of experience) will be included in the analysis models. Propensity scores will be used to correct for bias that could be introduced by selection bias. In this calculation, variables that are not considered as dependent variables or confounders of interest are used to predict the chance that a patient is included in either the CSC or the CAU group, using logistic regression analysis. This can be considered an appropriate procedure for cluster randomized trials [43].

Direct and indirect costs of the interventions will be reported. The results of the cost and QALY analyses will be presented as mean values with standard errors. Cost-effectiveness and cost-utility analyses will be presented in incremental cost-effectiveness ratios. The uncertainty will be assessed using bootstrapping [92] and acceptability curves will be presented. As principled methods (e.g. multiple imputation) take into account the special characteristics of cost data that affect their analysis, a principled method for dealing with missing data will be applied to our economic evaluation [93].

### Ethical principles

The study protocol has been approved by the Medical Ethical Committee of the VU University Medical Centre at April 29, 2008 and by the Medical Ethics Committee of the Leiden University Medical Centre at October 2^nd ^2008.

## Discussion

The presumed poor quality of care in the primary care setting for such prevalent, disabling and costly disorders as PD and GAD set the basis for this study. The aims of this study are to improve the quality of care for these patients with an acceptable increase in costs.

This study is the first in which a stepped care approach is incorporated in a collaborative care intervention for the treatment of anxiety disorders in primary care. It is also the first study, to our knowledge, that evaluates the cost-effectiveness of such an intervention outside the United States. Effective elements of other studies have been brought together in the protocol of this study.

A strength of this study is its pragmatic design. There is a minimum of exclusion criteria. Furthermore, care is provided by health care professionals from the field, unlike in other studies evaluating collaborative care [36-38]. Consequently, the results of this study may be generalized to naturalistic health care settings with a comparable primary health care system and will be easy to implement into practice.

## Abbreviations

PD: panic disorder; GAD: generalized anxiety disorder; GP: general practitioner; PCP: primary care practice; CP: consultant psychiatrist; CM: care manager; CSC: collaborative stepped care; CAU: care as usual; CBT: cognitive behavioral therapy.

## Competing interests

The authors declare that they have no competing interests.

## Authors' contributions

CFC is the principle investigator, she wrote the design of the study, participated in the development of the CBT protocol, took part in the training for psychiatrists and in writing this article. AvB and HvM contributed to the design of the study and the development of the CBT protocol, supervised the training of GPs and co-authored this article. AvB also participated in training the psychiatrists. MWMdW and WJJA contributed to the recruitment of GPs and gave advice on writing the article, WJJA also participated in the design of the study. PHS participated in the design of the study, developing the CBT protocol and co-authored the article. LH contributed to the design of the economic evaluation and co-authored the article. HA performed the randomization procedure and participated in describing the statistical analyses in this article. AM participated in the preparation phase of the study, assisted in the training and supervision of GPs, psychiatrists and care managers, contributed to the development of the CBT protocol and wrote this article.

## Pre-publication history

The pre-publication history for this paper can be accessed here:



## References

[B1] Buist-Bouwman MA, de Graaf R, Vollebergh WA, Alonso J, Bruffaerts R, Ormel J (2006). Functional disability of mental disorders and comparison with physical disorders: a study among the general population of six European countries. Acta Psychiatr Scand.

[B2] Kessler RC, Greenberg PE, Mickelson KD, Meneades LM, Wang PS (2001). The effects of chronic medical conditions on work loss and work cutback. J Occup Environ Med.

[B3] Cramer V, Torgersen S, Kringlen E (2005). Quality of life and anxiety disorders: a population study. J Nerv Ment Dis.

[B4] Andlin-Sobocki P, Wittchen HU (2005). Cost of anxiety disorders in Europe. Eur J Neurol.

[B5] Wittchen HU, Kessler RC, Beesdo K, Krause P, Hofler M, Hoyer J (2002). Generalized anxiety and depression in primary care: prevalence, recognition, and management. J Clin Psychiatry.

[B6] Young AS, Klap R, Sherbourne CD, Wells KB (2001). The quality of care for depressive and anxiety disorders in the United States. Arch Gen Psychiatry.

[B7] Kroenke K, Spitzer RL, Williams JB, Monahan PO, Lowe B (2007). Anxiety disorders in primary care: prevalence, impairment, comorbidity, and detection. Ann Intern Med.

[B8] Lieb R, Becker E, Altamura C (2005). The epidemiology of generalized anxiety disorder in Europe. Eur Neuropsychopharmacol.

[B9] Roy-Byrne PP, Wagner A (2004). Primary care perspectives on generalized anxiety disorder. J Clin Psychiatry.

[B10] Roy-Byrne PP, Wagner AW, Schraufnagel TJ (2005). Understanding and treating panic disorder in the primary care setting. J Clin Psychiatry.

[B11] Prins MA, Verhaak PF, Bensing JM, Van Der MK (2008). Health beliefs and perceived need for mental health care of anxiety and depression--the patients' perspective explored. Clin Psychol Rev.

[B12] Otto MW, Deveney C (2005). Cognitive-behavioral therapy and the treatment of panic disorder: efficacy and strategies. J Clin Psychiatry.

[B13] Gorman JM (2003). Treating generalized anxiety disorder. J Clin Psychiatry.

[B14] NHG (2004). NHG Standaard Angststoornis [Dutch college of general practitioners, Practical guideline Anxiety disorder]. Retrieved March 2, 2007, from Nederlands Huisartsen Genootschap (NHG). http://nhg.artsennet.nl/kenniscentrum/k_richtlijnen/k_nhgstandaarden/Samenvattingskaartje-NHGStandaard/M62_svk.htm.

[B15] The National Institute for Health and Clinical Excellence (NICE) (2007). CG22 Anxiety: Quick reference guide (amended) (2007, December 3). Anxiety: management of anxiety (panic disorder, with or without agoraphobia, and generalised anxiety disorder) in adults in primary, secondary and community care. Clinical guideline 22 (amended). Issue date: December 2004, with amendments April 2007. http://www.nice.org.uk/nicemedia/pdf/cg022fullguideline.pdf.

[B16] Andrews G, Oakley-Browne M, Castle D, Judd F, Baillie A (2003). Summary of guideline for the treatment of panic disorder and agoraphobia. Australasian Psychiatry.

[B17] Stein MB, Sherbourne CD, Craske MG, Means-Christensen A, Bystritsky A, Katon W (2004). Quality of care for primary care patients with anxiety disorders. Am J Psychiatry.

[B18] Fernandez A, Haro JM, Martinez-Alonso M, Demyttenaere K, Brugha TS, Autonell J (2007). Treatment adequacy for anxiety and depressive disorders in six European countries. Br J Psychiatry.

[B19] Wittchen HU, Hoyer J (2001). Generalized anxiety disorder: nature and course. J Clin Psychiatry.

[B20] Weiller E, Bisserbe JC, Maier W, Lecrubier Y (1998). Prevalence and recognition of anxiety syndromes in five European primary care settings. A report from the WHO study on Psychological Problems in General Health Care. Br J Psychiatry Suppl.

[B21] Jackson JL, Passamonti M, Kroenke K (2007). Outcome and impact of mental disorders in primary care at 5 years. Psychosom Med.

[B22] Alonso J, Buron A, Bruffaerts R, He Y, Posada-Villa J, Lepine JP (2008). Association of perceived stigma and mood and anxiety disorders: results from the World Mental Health Surveys. Acta Psychiatr Scand.

[B23] Mechanic D (2007). Barriers to help-seeking, detection, and adequate treatment for anxiety and mood disorders: implications for health care policy. J Clin Psychiatry.

[B24] Nutting PA, Rost K, Smith J, Werner JJ, Elliot C (2000). Competing demands from physical problems: effect on initiating and completing depression care over 6 months. Arch Fam Med.

[B25] Ormel J, Koeter MW, Brink Wvd, Willige Gvd (1991). Recognition, management, and course of anxiety and depression in general practice. Archives of General Psychiatry.

[B26] Rollman BL, Hanusa BH, Lowe HJ, Gilbert T, Kapoor WN, Schulberg HC (2002). A randomized trial using computerized decision support to improve treatment of major depression in primary care. J Gen Intern Med.

[B27] Mathias SD, Fifer SK, Mazonson PD, Lubeck DP, Buesching DP, Patrick DL (1994). Necessary but not sufficient: the effect of screening and feedback on outcomes of primary care patients with untreated anxiety. J Gen Intern Med.

[B28] Schulberg HC, Block MR, Madonia MJ, Scott CP, Lave JR, Rodriguez E (1997). The 'usual care' of major depression in primary care practice. Arch Fam Med.

[B29] van Boeijen CA, van Oppen P, van Balkom AJ, Visser S, Kempe PT, Blankenstein N (2005). Treatment of anxiety disorders in primary care practice: a randomised controlled trial. Br J Gen Pract.

[B30] Yonkers KA, Bruce SE, Dyck IR, Keller MB (2003). Chronicity, relapse, and illness--course of panic disorder, social phobia, and generalized anxiety disorder: findings in men and women from 8 years of follow-up. Depress Anxiety.

[B31] Wagner EH, Austin BT, Von KM (1996). Organizing care for patients with chronic illness. Milbank Q.

[B32] Katon W, Von Korff M, Lin E, Unutzer J, Simon G, Walker E (1997). Population-based care of depression: effective disease management strategies to decrease prevalence. Gen Hosp Psychiatry.

[B33] Katon W, Von KM, Lin E, Simon G (2001). Rethinking practitioner roles in chronic illness: the specialist, primary care physician, and the practice nurse. Gen Hosp Psychiatry.

[B34] Bower P, Gilbody S, Richards D, Fletcher J, Sutton A (2006). Collaborative care for depression in primary care: Making sense of a complex intervention: systematic review and meta-regression. Br J Psychiatry.

[B35] Gilbody S, Bower P, Fletcher J, Richards D, Sutton AJ (2006). Collaborative care for depression: a cumulative meta-analysis and review of longer-term outcomes. Arch Intern Med.

[B36] Roy-Byrne PP, Katon W, Cowley DS, Russo J (2001). A randomized effectiveness trial of collaborative care for patients with panic disorder in primary care. Arch Gen Psychiatry.

[B37] Rollman BL, Belnap BH, Mazumdar S, Houck PR, Zhu F, Gardner W (2005). A randomized trial to improve the quality of treatment for panic and generalized anxiety disorders in primary care. Arch Gen Psychiatry.

[B38] Roy-Byrne PP, Craske MG, Stein MB, Sullivan G, Bystritsky A, Katon W (2005). A randomized effectiveness trial of cognitive-behavioral therapy and medication for primary care panic disorder. Arch Gen Psychiatry.

[B39] Smolders M, Laurant M, Roberge P, Van Balkom A, Van Rijswijk E, Bower P (2008). Knowledge transfer and improvement of primary and ambulatory care for patients with anxiety. Can J Psychiatry.

[B40] Katon WJ, Roy-Byrne P, Russo J, Cowley D (2002). Cost-effectiveness and cost offset of a collaborative care intervention for primary care patients with panic disorder. Arch Gen Psychiatry.

[B41] Katon W, Russo J, Sherbourne C, Stein MB, Craske M, Fan MY (2006). Incremental cost-effectiveness of a collaborative care intervention for panic disorder. Psychol Med.

[B42] de Jong SJ, van Steenbergen-Weijenburg KM, Huijbregts KM, Vlasveld M, Marwijk HWJ, Beekman A, van der Feltz-Cornelis CM (2009). The depression initiative. Description of a collaborative care model for depression and of the factors influencing its implementation in the primary care setting in the Netherlands. Int J Integr Care.

[B43] Feltz-Cornelis CM Van der, van Oppen P, Adèr H, van Dyck R (2006). Randomised Controlled Trial of a Collaborative Care Model with Psychiatric Consultation for Persistent Medically Unexplained Symptoms in General Practice. Psychother Psychosom.

[B44] Feltz-Cornelis CM Van der, Ader HJ (2000). Randomization in psychiatric intervention research in the general practice setting. International Journal of Methods in Psychiatric Research.

[B45] (2001). Diagnostic and statistical manual of mental disorders, (DSM-IV).

[B46] Spitzer RL, Kroenke K, Williams JB (1999). Validation and utility of a self-report version of PRIME-MD: the PHQ primary care study. Primary Care Evaluation of Mental Disorders. Patient Health Questionnaire. JAMA.

[B47] Diez-Quevedo C, Rangil T, Sanchez-Planell L, Kroenke K, Spitzer RL (2001). Validation and utility of the patient health questionnaire in diagnosing mental disorders in 1003 general hospital Spanish inpatients. Psychosom Med.

[B48] Farrin A, Russell I, Torgerson D, Underwood M (2005). Differential recruitment in a cluster randomized trial in primary care: the experience of the UK back pain, exercise, active management and manipulation (UK BEAM) feasibility study. Clin Trials.

[B49] Lowe B, Grafe K, Zipfel S, Spitzer RL, Herrmann-Lingen C, Witte S (2003). Detecting panic disorder in medical and psychosomatic outpatients: comparative validation of the Hospital Anxiety and Depression Scale, the Patient Health Questionnaire, a screening question, and physicians' diagnosis. J Psychosom Res.

[B50] Diez-Quevedo C, Rangil T, Sanchez-Planell L, Kroenke K, Spitzer RL (2001). Validation and utility of the patient health questionnaire in diagnosing mental disorders in 1003 general hospital Spanish inpatients. Psychosom Med.

[B51] van Vliet IM, Leroy H, van Megen HJM (2000). De MINI-Internationaal neuropsychiatrisch interview: een kort gestructureerd diagnostisch interview voor DSM-IV en ICD-10 psychiatrische stoornissen.

[B52] Sheehan DV, Lecrubier Y, Sheehan KH, Amorim P, Janavs J, Weiller E (1998). The Mini-International Neuropsychiatric Interview (M.I.N.I.): the development and validation of a structured diagnostic psychiatric interview for DSM-IV and ICD-10. J Clin Psychiatry.

[B53] Wells KB, Burnam MA, Leake B, Robins LN (1988). Agreement between face-to-face and telephone-administered versions of the depression section of the NIMH Diagnostic Interview Schedule. J Psychiatr Res.

[B54] Cohen J (1988). Statistical power analysis for the behavioral science.

[B55] Penninx BW, Beekman AT, Smit JH, Zitman FG, Nolen WA, Spinhoven P (2008). The Netherlands Study of Depression and Anxiety (NESDA): rationale, objectives and methods. Int J Methods Psychiatr Res.

[B56] Adams G, Gulliford MC, Ukoumunne OC, Eldridge S, Chinn S, Campbell MJ (2004). Patterns of intra-cluster correlation from primary care research to inform study design and analysis. J Clin Epidemiol.

[B57] Gilbody S, Bower P, Torgerson D, Richards D (2008). Cluster randomized trials produced similar results to individually randomized trials in a meta-analysis of enhanced care for depression. J Clin Epidemiol.

[B58] Cosby RH, Howard M, Kaczorowski J, Willan AR, Sellors JW (2003). Randomizing patients by family practice: sample size estimation, intracluster correlation and data analysis. Fam Pract.

[B59] van Boeijen CA (2006). Feasibility and efficacy of treatment for anxiety in primary care. Dissertation.

[B60] Beck AT, Epstein N, Brown G, Steer RA (1988). An inventory for measuring clinical anxiety: psychometric properties. J Consult Clin Psychol.

[B61] van Boeijen CA, van Balkom AJ, van OP, Blankenstein N, Cherpanath A, van DR (2005). Efficacy of self-help manuals for anxiety disorders in primary care: a systematic review. Fam Pract.

[B62] van Boeijen CA (2007). Handboek Begeleide Zelfhulp: Overwinnen van angstklachten. (*Handbook guided self help: Overcoming anxiety*). Apeldoorn.

[B63] van Balkom AJLM, Nauta CME, akker A (1995). Metaanalyses on the treatment of panic disorder with agoraphobia: Review and reexamination. Clinical Psychology and Psychotherapy.

[B64] Hunot V, Churchill R, Silva dL, Teixeira V (2007). Psychological therapies for generalised anxiety disorder. Cochrane Database Syst Rev.

[B65] Clark DM, Hawton K, Salkovskis PM, Clark DM (1989). Anxiety states: Panic and generalized anxiety. Cognitive behaviour therapy for psychiatric problems: A practical guide.

[B66] Wells A (1997). Cognitive therapy of anxiety disorders: A practice manual and conceptual guide.

[B67] Wells A (2007). Cognition about cognition: Metacognitive therapy and change in generalized anxiety disorder and social phobia. Cognitive and Behavioral Practice.

[B68] Terluin B, Van heest FB, Meer K Van der, Neomagus GJH, Hekman J, Aulbers LPJ (2004). NHG-Standaard Angststoornissen. Huisarts en Wetenschap.

[B69] Feltz-Cornelis CM van der (2002). Psychiatric consultation for patients with somatoform disorder in general practice.

[B70] Ferguson RJ (2000). Using the Beck Anxiety Inventory in Primary Care. Maruish, M E Handbook of psychological assessments in primary care settings.

[B71] Jacobson NS, Truax P (1991). Clinical significance: a statistical approach to defining meaningful change in psychotherapy research. J Consult Clin Psychol.

[B72] Norman SB, Cissell SH, Means-Christensen AJ, Stein MB (2006). Development and validation of an Overall Anxiety Severity And Impairment Scale (OASIS). Depress Anxiety.

[B73] Campbell-Sills L, Norman SB, Craske MG, Sullivan G, Lang AJ, Chavira DA, Bystritsky A, Sherbourne C, Roy-Byrne P, Stein MB (2008). Validation of a brief measure of anxiety-related severity and impairment: The Overall Anxiety Severity and Impairment Scale (OASIS). J Affect Disord.

[B74] Hemert AM (2003). Lichamelijke Klachten Vragenlijst.

[B75] Speckens AE, Spinhoven P, Sloekers PP, Bolk JH, van Hemert AM (1996). A validation study of the Whitely Index, the Illness Attitude Scales, and the Somatosensory Amplification Scale in general medical and general practice patients. J Psychosom Res.

[B76] Speckens AE, Van Hemert AM, Spinhoven P, Bolk JH (1996). The diagnostic and prognostic significance of the Whitely Index, the Illness Attitude Scales and the Somatosensory Amplification Scale. Psychol Med.

[B77] Euroqol group (1995). Eq-5D user guide.

[B78] Ware JE, Sherbourne CD (1992). The MOS 36-Item Short-Form Health Survey (SF-36). Medical Care.

[B79] Roy-Byrne P, Stein MB, Russo J, Craske M, Katon W, Sullivan G (2005). Medical illness and response to treatment in primary care panic disorder. Gen Hosp Psychiatry.

[B80] Kroenke K, Spitzer RL, Williams JB (2001). The PHQ-9: validity of a brief depression severity measure. J Gen Intern Med.

[B81] van Balkom AJ, van Boeijen CA, Boeke AJ, van OP, Kempe PT, van DR (2008). Comorbid depression, but not comorbid anxiety disorders, predicts poor outcome in anxiety disorders. Depress Anxiety.

[B82] Schreurs PJG, Willige G van de, Brosschot JF, Tellegen JF, Grauw GMH (2007). Handleiding Utrechtse Copinglijst UCL *[Utrecht Coping Questionnaire]*. Human Reproduction.

[B83] Vollrath M, Alnaes R, Torgersen S (1996). Differential effects of coping in mental disorders: a prospective study in psychiatric outpatients. J Clin Psychol.

[B84] Lamers LM, McDonnell J, Stalmeier PF (2006). The Dutch tariff: results and arguments for an effective design for national EQ-5D valuation studies. Health Econ.

[B85] Hakkaart-van Roijen L (2002). Manual Trimbos/iMTA questionnaire for costs associated with psychiatric illness (in Dutch).

[B86] Hakkaart-van Roijen L, van Straten A, Al M, Rutten F, Donker M (2006). Cost-utility of brief psychological treatment for depression and anxiety. Br J Psychiatry.

[B87] Oostenbrink JB, Bouwmans CAM, Koopmanschap MA (2004). Handleiding voor kostenonderzoek, methoden en standaardkostprijzen voor economische evaluaties in de gezondheidszorg (Manual for Research on Costs, Methods and Standardized Cost Prices for Economic Evaluation in Health Care).

[B88] van Roijen L, Essink-Bot ML, Koopmanschap MA (1996). Labor and health status in economic evaluation of health care. The Health and Labor Questionnaire. Int J Technol Assess Health Care.

[B89] van Dam QD, Spijker A, Arends LR, Hakkaart-Roijen L, Donker MCH, Trijsburg (1998). Costeffectiveness of psychotherapy: A feasiblity study (in Dutch). J Pychotherapy.

[B90] Koopmanschap MA, Rutten FF, van Ineveld BM, van Roijen L (1995). The friction cost method for measuring indirect costs of disease. J Health Econ.

[B91] Twisk JWR (2006). Applied Multilevel Analysis.

[B92] van Hout BA, Al MJ, Gordon GS, Rutten FF (1994). Costs, effects and C/E-ratios alongside a clinical trial. Health Econ.

[B93] Oostenbrink JB, Al MJ, Rutten-van Molken MP (2003). Methods to analyse cost data of patients who withdraw in a clinical trial setting. Pharmacoeconomics.

